# A Review of Outcomes of Descemet Membrane Endothelial Keratoplasty and Descemet Stripping Automated Endothelial Keratoplasty Interventions in Patients with Pre-Existing Glaucoma

**DOI:** 10.3390/jcm14103534

**Published:** 2025-05-18

**Authors:** Keya Jafari, Zahra Ashena, Magdalena Niestrata

**Affiliations:** 1 Moorfields Eye Hospital, London EC1V 2PD, UK; keya.jafari@nhs.net; 2 Queen’s Hospital, Barking, Havering and Redbridge University Hospitals NHS Trust, London RM7 0AG, UK; 3 East Suffolk and North Essex Foundation Trust, Colchester CO4 5JR, UK; m.niestrata@nhs.net

**Keywords:** endothelial keratoplasty, DMEK, DSAEK, glaucoma, graft survival

## Abstract

Glaucoma is known to impair the function of corneal endothelial cells for various reasons, which increases the likelihood of patients with glaucoma requiring endothelial keratoplasty. Among the techniques available, Descemet membrane endothelial keratoplasty (DMEK) and Descemet stripping automated endothelial keratoplasty (DSAEK) each present unique challenges, particularly for those with a history of glaucoma surgery. We conducted a literature review to evaluate the outcomes of DMEK and DSAEK in glaucoma patients, focusing on factors such as visual prognosis, graft survival, glaucoma exacerbations, and any necessary surgical modifications. The findings indicate that DMEK tends to provide better visual outcomes compared to DSAEK, with a lower rate of steroid responders due to a shorter postoperative steroid regimen. While DMEK has shown a higher incidence of graft detachment and a lower rate of graft rejection, compared to DSAEK, in the general population, the specific data regarding these outcomes in glaucoma patients remain scarce in the existing literature. Overall, the survival rates of both grafting techniques do not show significant differences within the glaucoma patient population. To draw more definitive conclusions about graft survival between the two methods, a greater number of comparative studies with longer follow-up periods is needed.

## 1. Introduction

The healthy human corneal endothelium maintains transparency of the cornea by preventing excess fluid accumulation within the cornea. Dysfunction of the corneal endothelium causes corneal swelling or oedema, which reduces corneal transparency and leads to blindness. Human corneal endothelial cells decline in number over time and have limited mitotic activity in vivo [1]. Due to the finite number of corneal endothelial cells, the human cornea endothelium has limited regenerative capacity, and repair occurs via stretching and migration of existing endothelial cells. Given this limited regenerative capacity, restoration of corneal endothelial function and corneal deturgescence requires surgical intervention in the form of endothelial keratoplasty, which involves transplantation of corneal endothelium.

Several pathologies can cause decline and dysfunction of corneal endothelial cells. Endothelial dystrophy is the leading indication for corneal endothelial keratoplasty, accounting for 51% of endothelial keratoplasties in the United States in 2020 [2]. Glaucoma is also a significant cause of a decline in endothelial cell count and morphology through various mechanisms [3], and can contribute to the need for endothelial keratoplasty.

Glaucoma is a progressive condition that can result in blindness. It is the leading cause of irreversible blindness worldwide, affecting 3.54% of people aged 40–80 years [4]. Glaucoma surgical interventions, medication use, intraocular pressure (IOP) fluctuations, and aqueous humour abnormalities are all exacerbating factors linked to corneal endothelial dysfunction [3]. Endothelial cell loss in glaucoma patients is well established [5,6,7] and corneal endothelial cell density is lower in patients with glaucoma than those without [6]. There are thought to be three main mechanisms for this. Firstly, increased IOP can affect the metabolic pumping mechanism of the corneal endothelium and can even cause loss of endothelial cells via direct compression of the corneal endothelium [3,6,8]. Additionally, glaucoma medications are known to decrease epithelial barrier function. Studies have shown that the active ingredients in topical glaucoma medications have little effect on the corneal endothelium [9]. However, preservatives in the drops, such as benzalkonium chloride, can potentially impact corneal endothelial function [10,11]. Finally, alterations to the corneal endothelial cell layer and trabecular meshwork can occur due to factors associated with the underlying aetiology of glaucoma, such as TFG-β [6].

Additionally, anterior segment procedures have been shown to induce endothelial cell damage and reduce endothelial cell density postoperatively [12]. Similarly, implantation of glaucoma drainage devices within the anterior chamber can induce progressive endothelial cell loss [13]. The exact mechanism of damage is unknown, though the cause is thought to be multifactorial [14,15,16]. Moreover, direct endothelial damage can occur secondary to a shallow or flat anterior chamber, which can frequently occur after trabeculectomy and glaucoma filtering procedures [17]. As well as contributing to the need for endothelial keratoplasty, these factors predispose surgical glaucoma patients to higher graft failure rates [18,19,20,21].

Endothelial keratoplasty involves the transplantation of corneal tissue to restore corneal endothelial function. Descemet stripping automated endothelial keratoplasty (DSAEK) and Descemet membrane endothelial keratoplasty (DMEK) have replaced penetrating keratoplasty (PK) as the standard of care for endothelial decompensation. They have shown to be highly successful procedures for corneal endothelial dysfunction, with excellent visual outcomes and graft survival [22]. However, endothelial keratoplasty in patients with pre-existing glaucoma poses challenges. It was noted in the Cornea Donor Study that a diagnosis of glaucoma or ocular hypertension was strongly associated with graft failure in PK [19]. The Collaborative Corneal Transplantation studies also showed higher graft failure rates in patients with glaucoma (48%) compared to non-glaucomatous causes of endothelial dysfunction (29%) [19]. This increased risk of graft failure persists in DSAEK procedures, specifically in patients with previous glaucoma surgery [18,19,20].

In addition to graft failure, endothelial keratoplasty in patients with existing glaucoma presents the additional risk of worsening glaucoma postoperatively. DMEK and DSAEK procedures can induce glaucoma in non-glaucomatous eyes and, indeed, worsen the progression of glaucoma and IOP control in patients with a pre-existing diagnosis [23].

Graft failure following DMEK procedures in patients with preoperative glaucoma is also increased compared to non-glaucomatous eyes [24,25,26,27]. Considering the complexity of the procedure in glaucomatous patients, it is important for surgeons to consider which of DMEK and DSAEK may be most suitable for patients with glaucoma and which holds the best chance of success. This review will outline and compare the current literature on outcomes of DSAEK and DMEK in patients with pre-existing glaucoma, with a focus on visual acuity and graft survival as well as any potential glaucoma exacerbation. Additionally, we will discuss the surgical considerations for DMEK and DSAEK procedures in glaucoma patients.

## 2. Methods of the Literature Review

A search was performed for all English publications on the PubMed database of studies published from 1 January 2006 (first report of human DMEK transplant) to 27 September 2024. The search terms were as follows: ((DMEK) OR (DSEK) OR (DSAEK) OR (endothelial keratoplasty) OR (EK) OR (Descemet membrane endothelial keratoplasty) OR (Descemet stripping endothelial keratoplasty)) AND ((Glaucoma) OR (Trabeculectomy) OR (Tube) OR (Shunt)). Case reports, clinical trials, observational studies, and randomised controlled trials were included. In total, 217 articles were identified and screened. Overall, 23 studies were relevant to the PICO. The outcomes reviewed were visual acuity, graft detachment, graft failure (primary and secondary), graft rejection, and IOP changes. Two studies compared DMEK to DSAEK in patients with pre-existing glaucoma.

Studies evaluating outcomes of endothelial keratoplasty in patients with any type of glaucoma (primary and secondary open-angle glaucoma; primary and secondary angle-closure glaucoma) were included. Studies that included patients with pre-existing glaucoma but did not report results on this subgroup of patients were excluded.

## 3. Results

### 3.1. Visual Acuity

Both DSAEK and DMEK have shown promising vision outcomes in patients with pre-existing glaucoma. Two papers directly compare visual acuity outcomes in DSAEK versus DMEK in patients with pre-existing glaucoma [28,29]. Lin et al. found that postoperative BCVA improved quicker in the DMEK group (BCVA > 20/40 at 12 months, 47% in DMEK versus 15% in DSAEK, *p* = 0.002) in their cohort of patients with surgical glaucoma [28]. Additionally, a significantly greater proportion of DMEK patients achieved BCVA > 20/40 at all follow-up time points (1 month *p* = 0.0005, 3 months *p* = 0.0002, 6 months *p* = 0.09, and 12 months *p* = 0.002) and DMEK eyes achieved better visual acuity in 1 month than the DSAEK group achieved in 1 year. There were no significant differences in preoperative BCVA in the two groups.

The second study, by Alshaker et al., found that postoperative BCVA was significantly better in the DMEK group at 6, 12, and 24 months (*p* < 0.001, *p* = 0.022 and *p* = 0.047) [29]. There was no significant difference in preoperative BCVA. Additionally, multivariate analysis accounting for preoperative demographics such as cup-to-disc ratio showed the only significant factor to be the type of endothelial keratoplasty (DMEK or DSAEK), potentially mitigating the variations in baseline vision potential secondary to glaucoma severity.

### 3.2. Graft Outcomes

#### 3.2.1. Graft Detachment

Graft detachment requiring rebubbling in surgical glaucoma patients who underwent DMEK is reported to occur in 15% to 74%, which is comparable to rates in non-glaucomatous eyes [27,30,31,32]. DSAEK patients with glaucoma drainage devices who require rebubbling range between 2% and 50% [33,34], which is also comparable to non-glaucomatous eyes [35] (Table 1).

Only two papers directly compared DMEK to DSAEK in patients with existing surgical glaucoma. Significant graft detachment was defined as any total or partial separation of the graft from the host cornea requiring rebubbling or repeat keratoplasty. The rates of graft detachment were comparable in both techniques, with no statistically significant difference between DMEK (22% and 31%) and DSAEK (9% and 20%) (Table 2) [28,29]. Detachment rates were not distinguished by whether the patients had a glaucoma drainage device (GDD) or not. However, Alshaker et al. conducted subgroup analysis on all patients with a GDD vs. those without and showed comparable rates of graft detachments in patients with a GDD (29.1%) to those without a GDD (23.5%), with no statistically significant difference [29]. The procedure type (DMEK vs. DSAEK) was not looked at in this analysis.

#### 3.2.2. Graft Rejection

DSAEK rejection rates in patients with pre-existing glaucoma range from 5.8% to 23% [33,36]. Comparably, DMEK rejection rates in patients with pre-existing glaucoma range from 0% to 21% [24,29,32,37] (Table 2).

#### 3.2.3. Graft Survival

Studies have reported 5-year graft survival rates in DSAEK patients with previous glaucoma surgery between 40 and 48%, compared to 90–94% in patients with medically managed glaucoma and 95–96% in non-glaucomatous eyes [18,38].

Similarly, surgical glaucoma patients who undergo DMEK have a higher rate of secondary graft failure. Secondary graft failure is reported to range from 10.3% to 47.1% of cases of DMEK in patients with surgical glaucoma [25,39]. This is compared to 2.4% in DMEK patients without glaucoma [39] (Table 2).

### 3.3. Exacerbation of Glaucoma

Exacerbation of glaucoma was defined as persistently raised postoperative IOP or a need for escalation in glaucoma therapy or surgery.

Overall, the reports of escalation of medical management of glaucoma in glaucoma patients undergoing DSAEK range from 4% to 47.4% of eyes [33,40] (Table 1).

Rates of exacerbation of glaucoma control post DMEK in patients with pre-existing surgical glaucoma range from 6.4% to 53% [26,29] (Table 1). Glaucoma control in patients with pre-existing medical glaucoma is also reported to worsen in around 21% to 55% of cases [24,32].

Comparably, in patients without glaucoma who underwent DMEK, reports show that elevated IOP (≥22–25 mmHg or IOP change of ≥10 mmHg from baseline) occurred in 6.5% to 23.3% of cases and the incidence of glaucoma in these patients was 2.7% to 6.6% [23,32,37,41].

Glaucoma patients undergoing DSAEK often need escalation in the medical management of their glaucoma. In patients with a history of glaucoma surgery, 24.5% to 38% required additional medical therapy and 7.8% to 19% required surgical glaucoma intervention [42,43]. The highest incidence of 47.4% comes from medically treated glaucoma patients in Wiaux et al.’s study [33], which looked at 213 eyes over an average of 11.9 months and found that 19.2% of all eyes developed an IOP of ≥25 across the 12 month follow-up period. In the medically treated glaucoma group, 47.4% had an IOP ≥ 25, and this was significantly higher than that in surgically treated glaucoma (11.5%) and patients without an existing history of glaucoma (18.3%). In keeping with this, a significantly greater percentage of patients in the medically treated glaucoma group required an increase in glaucoma drops (47.4%) compared to the surgically treated (28.8%) and non-glaucoma groups (21.1%), indicating a greater risk of progression of glaucoma in DSAEKs on patients with medical glaucoma. Four patients required subsequent glaucoma surgery to control their IOP, three (5.8%) from the surgically treated glaucoma group and one (5.3%) from the medically treated glaucoma group, with no statistical difference. Kang et al. [44] looked at IOP control post DSAEK procedures in 85 eyes with glaucoma drainage devices, and defined reduced IOP control as a patient where initiation or an increase in the number of glaucoma drops was required, steroid eye drops were reduced, or additional glaucoma surgical procedure were required. They report similar results, with 24.7% of eyes requiring escalation in IOP control. In a similar report on 102 eyes with surgical glaucoma, Kang et al. [42] report an escalation in the number of topical glaucoma medications in 24.5% of eyes and additional glaucoma surgery in 7.8% of eyes. They do not, however, differentiate the patients by type of surgical glaucoma in their results (GDD vs. trabeculectomy). Maeir et al. [45] report that 33.3% of their 15 DSAEK patients with preoperative glaucoma diagnosis required escalation in glaucoma medical or surgical management across a mean 14.4 month follow-up period.

Similarly, glaucoma control is also reported to worsen in patients undergoing DMEK. However, some studies report no significant association between pre-existing medically treated glaucoma and postoperative ocular hypertension [23,24,37,41,46,47].

In the two studies that compare DMEK to DSAEK in glaucoma patients, incidences of raised IOP were 14.6% and 53% in surgical glaucoma patients who underwent DMEK and 17.1% and 39% in patients who underwent DSAEK, with no statistical difference between the procedures [28,29]. Lin et al. defined raised IOP as an increase in IOP of >8 mmHg compared to preoperative IOP lasting more than 1 week or by the need for further glaucoma surgery after postoperative week 1 [28]. In their DMEK group, five patients (11%) required surgical glaucoma management of their raised IOP, compared to two patients (4%) in the DSAEK group.

**Table 1:** Studies evaluating outcomes after DMEK and DSAEK surgery in patients with prior glaucoma (NG = no glaucoma, MG = medically treated glaucoma, SG = surgical glaucoma, mins = minutes; m = months; f/up = follow-up).

Author	Operation	Mean F/Up (m)	No. of Eyes			Primary Graft Failure (%)			Secondary Graft Failure (%)			Graft Rejection (%)			Graft Detachment/Need for Rebubbling (%)			Endothelial Cell Loss (%)		
			NG	MG	SG	NG	MG	SG	NG	MG	SG	NG	MG	SG	NG	MG	SG	NG	MG	SG
DMEK vs. DSAEK																				
Lin et al. (2019) [28]	DMEK	17	0	0	46			2			0			4			22			
	DSAEK	21	0	0	46			2			17			9			9			
Alshaker et al. (2021) [29]	DMEK	30	0	0	48			15			0			21			31			48% (12 m); 55% (24 m); 57% (36 m)
	DSAEK	34	0	0	41			15			8			20			22			53% (12 m); 60% (24 m); 59% (36 m)
DMEK																				
			NG	MG	SG	NG	MG	SG	NG	MG	SG	NG	MG	SG	NG	MG	SG	NG	MG	SG
Naveiras et al. (2012) [37]	DMEK	22	247	28		0	0	0	0	0	0	0	0						at 6 m: between 8 and 32% ECD	
Heindl et al. (2013) [48]	DMEK	12	0	0	2												50			
Marianne et al. (2014) [46]	DMEK	12	296	29								1						20–41% ECL		
Aravena et al. (2016) [32]	DMEK	10	60	14	34	10	0	0	0	0	0	1	0	0	23	50	15	33	30	45
Birbal (2019) [27]	DMEK	19	0	0	23						9			9			74			71% (at 12 m)
Bonnet et al. (2020) [24]	DMEK	38	41	11	38	0	0	0	2	9	32	15	0	16	20	18	16	~45% (last f/up > 48 m)	~48% (last f/up > 48 m)	~64% (last f/up > 48 m)
Sorkin et al. (2020) [25]	DMEK	38 (study group), 34 (control group)			32 (24 GDD, 7 GDD + Trab, 1 GDD + MIGS)	12	0	16	0		47	2		20	37		40	52% (48 m)		74% (48 m)
Boutin et al. (2021) [39]	DMEK	15			27			4			10			17			24			37% (6 m); 51% (12 m)
Maeir et al. (2022) [49]	DMEK	26	0	109	41 (21 trab, 10 GDD; 10 other)		3			16.6% at 36 m			9.2% at 36 m			23			23% (12 m) 32% (36 m)	
Schrittenlocher et al. (2022) [26]	DMEK	36	0	0	66 (27 GDD, 39 Trab)			7.4% in GDD; 0% in Trab			56% in GDD; 35.9% in Trab			14.8% in GDD; 7.7% in Trab			18.5% in GDD; 35.95% in Trab			27% (24 m) in GDD; 35% (24 m) in Trab
DSEK/DSAEK																				
			NG	MG	SG	NG	MG	SG	NG	MG	SG	NG	MG	SG	NG	MG	SG	NG	MG	SG
Phillips et al. (2010) [50]	DSAEK	1	431		28	0		0												
Wandling et al. (2012) [51]	DSAEK	29	22	5	7															
Iverson et al. (2015) [36]	DSEK	15		17	13					29	77% (at last f/up)		18	23		18	39			
Iverson et al. (2018) [52]	DSEK	15		56						38% (last f/up, mean 9.5 m)	50% (last f/up, mean 5.8 m)		8	0			23	25		
Kang et al. (2016) [44]	DSAEK	29			102			2			23			9			36			
Kaleem (2017) [40]	DSAEK		235	144																
Aldave (2014) [53]	DSEK	21	299	50	113	3		4	3		16	7		13	12		14			
Wiaux (2011) [33]	DSEK	12	142	19	52	4		5	2		7	5		6	15		13			
Kang et al. (2019) [44]	DSEK	37			85			2			32			9			32			

**Table 2:** Studies evaluating exacerbation of glaucoma after DMEK and DSAEK surgery in patients with prior glaucoma (NG = no glaucoma, MG = medically treated glaucoma, SG = surgical glaucoma).

Author	Study Type	Operation	Mean F/Up (Months)	No. of Eyes			Graft Attachment Technique	Exacerbation of Glaucoma/Incidence of Postoperative Raised IOP			
				NG	MG	SG		NG	MG	SG	*p*
DMEK vs. DSAEK											
Lin et al. (2019) [28]	Case-matched retrospective comparative case series	DMEK	17	0	0	46	Complete air fill for 8–10 min, then 80–90% fill left in place with air or 20% SF6.			53%	0.66
		DSAEK	21	0	0	46	Complete air fill for 8–10 min, then 5–7 mm bubble left in place with air or 20% SF6.			39%	
Alshaker et al. (2021) [29]	Retrospective comparative study	DMEK	30	0	0	48	Complete air fill, the air bubble was then reduced to a diameter slightly larger than that of the graft.			15%	0.768
		DSAEK	34	0	0	41	Complete air fill for 10 min, and then part of the air removed and replaced with BSS.			17%	
DMEK											
				NG	MG	SG		NG	MG	SG	
Naveiras et al. (2012) [37]	Nonrandomised prospective cohort study	DMEK	22	247	28		Complete air fill for 45–60 min, then released to 50% air fill.	5%	25%		
Heindl et al. (2013) [48]	Retrospective observational case series	DMEK	12	0	0	2	Complete air fill for 5 min, then released to 20% air fill.			1 (50%)	
Marianne et al. (2014) [46]	Prospective, randomised, open-label controlled trial	DMEK	12	296	29				55%		
Aravena et al. (2016) [32]	Retrospective observational study	DMEK	10	60	14	34		23%	21%	24%	
Birbal (2019) [27]	Retrospective observational study	DMEK	19	0	0	23	Complete air fill for >60 min and in most eyes the air bubble was not reduced.			9%	
Bonnet et al. (2020) [24]	Retrospective, noncomparative case series	DMEK	38	41	11	38	Complete air fill for 10 min, with a 90% air fill left in place after the procedure in most cases.	34%	55%	25%	
Sorkin et al. (2020) [25]	Retrospective observational study	DMEK	38 (study group), 34 (control group)			32 (24 GDD, 7 GDD + Trab, 1 GDD + MIGS)	Complete air fill, the air bubble was then reduced to a diameter slightly larger than that of the graft.			8%	
Maeir et al. (2022) [49]	Retrospective observational case series analysis	DMEK	26	0	109	41 (21 trab, 10 GDD, 10 other)	Complete air fill for >60 min and in most eyes the air bubble was not reduced.		54%		
Schrittenlocher et al. (2022) [26]	Clinical retrospective review	DMEK	36	0	0	66 (27 GDD, 39 trab)	Complete air fill or sulphur hexafluoride 20% (SF6 20%) fill. Decision was independent of patient-related factors; procedures after July 2015 used SF6 20%.			7.4% in GDD; 6.4% in Trab (steroid responses)	
DESK/DSAEK											
				NG	MG	SG		NG	MG	SG	
Vajaranant et al. (2009) [43]	Retrospective case review	DSEK	NA	315	64	21		35%	45%	43%	
Phillips et al. (2010) [50]	Retrospective case review	DSAEK	1 month	431		28	Complete air fill of anterior chamber for 10 min, dilating drops added at this point. A final air bubble of only 8 or 9 mm was then placed into the anterior chamber.	17%		14%	
Wandling et al. (2012) [51]	Retrospective case review	DSAEK	29	22	5	7		41%			
Iverson et al. (2015) [36]	Retrospective case review	DSEK	15		17	13	Complete air fill for 10 min. Fluid–air exchange was then performed, and patient left with 80% air fill of the anterior chamber.		53%	62%	
Iverson et al. (2018) [52]	Retrospective case review	DSEK	15		56		Complete air fill for 10 min. Fluid-air exchange was then performed, and patient left with 80% air fill of the anterior chamber.		77%	42%	
Kang et al. (2016) [44]	Retrospective observational review	DSAEK	29.1			102	Complete air fill for 10 min. Fluid–air exchange was then performed, and patient left with 80% air fill of the anterior chamber.			24.5% increase in medical management; 7.8% required further surgical management	
Sharma (2015) [54]	Retrospective cohort study	DSEK	6					23% IOP elevation	20% IOP elevation		
Kaleem (2017) [40]	Retrospective observational review	DSAEK		235	144			3%	4%		
Aldave (2014) [53]	Retrospective observational review	DSEK	21	299	50	113		20%	41%	24%	
Wiaux (2011) [33]	Retrospective case review	DSEK	12	142	19	52		21%	53%	35%	
Kang et al. (2019) [44]	Retrospective case review	DSEK	37			85				25%	

## 4. Discussion

### 4.1. Visual Acuity

Patients with pre-existing glaucoma often have limited vision potential regardless of any corneal pathology. It is, therefore, difficult to accurately compare pre- and postop visual improvement after endothelial keratoplasty, as a significant proportion of visual loss in the majority of these patients can be attributed to their glaucoma, which the endothelial keratoplasty would not rectify.

The thinner graft and decreased interface backscatter are thought to contribute to better visual outcomes in DMEK compared to DSAEK [55,56]. It is well established that DMEK produces better visual outcomes compared to DSAEK in non-complex eyes. Whether this advantage translates to more complex cases, such as surgical glaucoma patients, remains debated [57,58,59].

Lin et al. report better visual outcomes in DMEK compared to DSAEK in patients with surgical glaucoma [28]. However, Lin et al.’s findings do not account for the variability in preoperative vision potential in their cohort of glaucomatous patients. Similarly, Alshaker et al. showed DMEK to have significantly better outcomes in their cohort of surgical glaucoma patients compared to DSAEK, at 6, 12, and 24 months [29]. The visual recovery in DMEK is also quicker [28]. This faster visual recovery and better BCVA mirrors existing data on DMEK compared to DSAEK visual outcomes in other cohorts [55,56,60], suggesting that this effect may persists even in complex cases.

Faster visual recovery and better visual acuity are especially impactful in patients with glaucoma, given their predisposition to functional visual deficits and significant field defects. A vision acuity of 20/40 or better, as achieved in 47% of the patients in Lin et al.’s study in surgical glaucoma patients, enables patients to achieve driving standards [61], recognise faces [62], and read effectively [63].

### 4.2. Graft Outcomes

#### 4.2.1. Graft Detachment

Patients with a GDD present a unique challenge in graft detachment due to the presence of a drainage device or trabeculectomy, which can enable air escape, resulting in a reduction in the air bubble, impacting graft adherence to the stromal bed. This can cause an increase in graft dislocations and detachments [64].

In studies not specific to glaucomatous eyes, graft detachment rates are higher in DMEK compared to DSAEK [60]. It is difficult to comment on whether this trend persists in glaucomatous eyes given the scarcity of literature, variability reported in studies, and lack of a controlled comparison. The most conclusive evidence we currently have by Lin et al. and Alshaker et al. reports comparable rates between the two procedures in glaucomatous eyes, regardless of having a GDD. Although Lin et al. found higher rates of rebubbling were required in DMEKs, this was not statistically significant. It may be that the rates of detachment and rebubbling are similar in DSAEK and DMEK in surgical glaucoma cases due to the increased complexity and lower tendency to maintain a high IOP for the 10 min full air fill period to avoid further optic nerve damage in these glaucomatous cases, resulting in equally high detachment or rebubbling rates in DSAEKs as in DMEKs.

#### 4.2.2. Graft Rejection

Patients with pre-existing glaucoma have higher rejection rates in DSAEK compared to those without the condition [18,33,53]. Price et al. looked at the outcomes of 598 DSAEK cases and found that eyes with pre-existing glaucoma or steroid-responsive ocular hypertension had 1.8 times the relative risk of graft rejection compared to eyes without glaucoma or ocular hypertension. The rejection episodes all occurred after a reduction in steroid drop use to combat postoperative increased IOP. This is of particular relevance as there is a decreased requirement for postoperative steroid drops in DMEK compared to DSAEK, which may reduce the risk of steroid-induced raised intraocular pressure and the need for early weaning of steroid drops postoperatively, which puts the graft at increased risk of rejection. Other studies have shown no statistical difference between rejection rates in DSAEK eyes with or without pre-existing glaucoma [18,53].

Theoretically, the thinner graft in a DMEK and the decreased amount of transplanted tissue incites less of an immune response and is thought to be responsible for a decreased rate of rejection in DMEKs [60,65,66]. Sorkin et al. found that DMEK patients with pre-existing glaucoma have a higher proportion of graft rejection (19.6%) compared to those without prior glaucoma surgery (2.3%) [25]. There was no significant difference in rejection rates in DMEK (4% and 20.8%) and DSAEKs (9% and 19.5%) in the two comparative studies on this cohort of patients with pre-existing surgical glaucoma [28,29]. The sample size in these studies was limited in each subgroup of DMEK and DSAEK, and considering the rarity of rejection rate in endothelial keratoplasty, more studies with larger cohorts are required to identify the true comparison of the rejection rate between these two techniques.

#### 4.2.3. Graft Survival

Corneal endothelial cell count progressively decreases in patients with glaucoma drainage devices, impacting graft survival after endothelial keratoplasty [25]. The cause of this is thought to be multifactorial. Firstly, glaucoma tube shunts can be directly traumatic to the corneal endothelium of the donor graft [67]. Though not specific to eyes that underwent EK, studies have shown that the closer the tube is positioned to the cornea, the greater the endothelial cell loss [68,69]. Secondly, tube implantation can disrupt the blood–aqueous barrier and increase inflammatory proteins in the aqueous humour, resulting in endothelial decompensation [65]. Additionally, drainage devices can influence the circulation patterns of aqueous humour, which adversely impacts endothelial cells [14,15].

More directly, endothelial keratoplasty in patients with a prior tube shunt can be impeded by the difficulty of optimally positioning the graft, leading to increased intraoperative manipulation of the graft, hence a reduced endothelial cell count and shortened graft survival [70]. The rates of secondary graft failure in eyes with previous glaucoma surgery, particularly in eyes with drainage devices, are therefore higher than in eyes without glaucoma [18,38,42,54]. Additionally, Anshu et al. demonstrated that graft failure rates increased with the number of glaucoma surgeries. Eyes with one prior glaucoma surgery were 9 times more likely to fail, and those with two or more prior surgeries were 27 times more likely to fail [18].

Lin et al. report a higher rate of secondary graft failure in DSAEK compared to DMEK in patients with surgical glaucoma at 1-year follow-up in their case-match comparative case series (17% vs. 0%, *p* = 0.06) [28]. This corresponds with data from other studies directly comparing DMEK and DSAEK, which report lower rejection and secondary failure rates, with increased graft survival in DMEK procedures, though not specific to patients with surgical glaucoma [60,66,71]. This trend may be due to several factors, including reduced immunogenicity from the absence of stromal tissue and subsequent lower rates of rejection as well as decreased postoperative interface inflammation in DMEKs.

However, over a longer follow-up period of 4 years, Alshaker et al. report equitable primary failure, endothelial cell loss, endothelial rejection, and secondary failure rates in both DMEK and DSAEK [29].

### 4.3. Exacerbation of Glaucoma

Endothelial keratoplasty can adversely affect the progression of glaucoma. There are multiple causes of IOP elevation after endothelial keratoplasty [Table 3]. IOP rise following DMEK and DSAEK can be categorised into early and late causes.

In the early perioperative phase, ocular hypertension can occur due to air/gas overfill, pupillary block, and retained viscoelastic [23,37,72,73,74,75,76]. Adequate air fill is required for a successful DMEK, as underfilling the eye can result in graft detachment—the most common complication of DMEKs [30,31]. In glaucomatous eyes, graft detachment and the need for rebubbling is primarily influenced by the presence of a trabeculectomy or tube shunt, which can make complete air fill challenging due to the egress of air into the trabeculectomy or tube shunt. To reduce graft detachment risk, it is therefore important to establish a good amount of air fill in such cases, which is accompanied by acute rises in IOP. Moreover, there are reported cases of postoperative IOP spike in patients with previously functioning trabeculectomy, in which the SF6 gas entered and blocked the bleb, acutely impairing its function and raising the IOP to 50 mmHg [77]. Close monitoring in the perioperative period is required to identify acutely raised IOP, particularly as it can present asymptomatically. As well as exacerbating glaucoma, fluctuations in IOP can result in early endothelial cell loss [3,47]. Additionally, migration of air bubbles can also rapidly increase the IOP by causing a pupillary block with iris bombe, reverse pupillary block with a concave iris, or migration of air behind the iris [75,78]. Theoretically, the risk of pupillary block glaucoma is higher in DMEK than in DSAEK due to the larger air or gas bubble typically used to fill the anterior chamber and support the graft in DMEKs. Prophylactic iridotomy/iridectomy is therefore recommended in most DMEK cases to prevent occurrences of pupil block.

Pupil block is reported to occur between 0% and 15.4% of cases in DMEK [23,27,37,74]. However, the 15.4% rate reported by Maier et al. occurred in the absence of placing a prophylactic surgical peripheral iridotomy/iridectomy (PI). Most DMEK studies that used prophylactic peripheral iridotomy/iridectomy report no incidences of pupillary block [30,37,78,79,80,81,82]. Comparably, DSAEK incidences of pupillary block are reported to be between 0% and 13.6% in patients [45,74].

Additionally, pupil block may be more likely to occur in eyes with pre-existing IOL implants, as opposed to in cases of combined procedures. Gonzalez et al. found significantly higher rates of full air fill at 1h postoperative slit lamp assessment in patients who had a combined DMEK + phaco IOL procedure [30%] compared to those who had just DMEK who were already pseudophakic (16%) [74]. However, all cases of full air fill in the combined cohort self-resolved and did not develop pupil block glaucoma or require air removal. They hypothesise that this is due to the phakic patients undergoing combined cases having more flexible zonular fibres than pseudophakic patients, enabling the air bubble to take on a more spherical shape and prevent air occlusion over a peripheral iridotomy. Further subgroup analysis would need to be performed on this cohort of patients in comparative studies to formalise a distinction.

Late increase in IOP is usually attributed to a steroid response, which typically occurs from 2 weeks postoperatively [46,73]. Other causes of late IOP elevations include blockage of the trabecular meshwork with inflammatory debris, formation of peripheral anterior synechiae, and damage to the angle [23,41].

Whilst most studies on endothelial keratoplasty in glaucoma patients comment on IOP changes postoperatively, few analyse glaucoma progression. In the studies that attempt to evaluate this outcome, worsening of glaucoma control is typically characterised by increased postoperative IOP or need for escalation in glaucoma therapy or surgery. Visual field testing is unreliable in this subset of patients due to poor vision preoperatively.

In the two studies that directly compare DMEK to DSAEK in surgical glaucoma patients, there is no mention of the number of eyes that required an escalation of glaucoma drops. Alshaker et al. [29] do not distinguish how many eyes within each group (DMEK vs. DSAEK) required surgical management; across both groups, three eyes required surgical management, five eyes were managed medically, and four eyes required no further intervention (patient request or blind eye).

In summary, DSAEK and DMEK are known to increase the incidence or progression of glaucoma and ocular hypertension postoperatively in both glaucoma naïve patients and pre-existing glaucoma patients. The extent of this effect appears to be greatest in patients with pre-existing medically managed glaucoma [33]. Existing studies do not provide enough data to compare IOP increase and glaucoma progression in DMEK vs. DSAEK in patients with glaucoma, though the two comparative studies that looked at these measures found no statistical difference between the two procedures in this cohort of patients.

## 5. Surgical Considerations for Clinicians

Endothelial keratoplasty (EK) in patients with previous glaucoma is technically challenging, particularly in patients with previous tube or trabeculectomy surgery. The presence of a shunt, iris defects, anterior synechiae, and hypotony all contribute to a more challenging environment in which to operate. DMEK grafts are more challenging to position and fix compared to DSAEK lamella. For this reason, some surgeons prefer DSAEK in eyes with complicated anterior chambers. To make DMEK feasible in these eyes, additional concurrent procedures are often required to recreate sufficient space in the anterior chamber before graft insertion [32].

Additional steps and technique modifications are often required for the success of DMEK and DSAEK in this high-risk group. Specific considerations include the following:

- Marinating sufficient anterior chamber air/gas fill to achieve graft attachment in the presence of iridectomy or drainage devices.
- Unfolding the graft when the anterior chamber anatomy is altered: When a large portion of a tube is far into the anterior chamber, it can interfere with insertion and unfolding of the graft especially in the case of DMEK, resulting in poorer graft survival [70]. Similarly, changes to the iris plane anatomy, such as in the case of a large peripheral iridectomy in trabeculectomy patients, can result in difficulties in unfolding a DMEK.

In view of the above, it is important to modify the surgical technique as well as the preoperative and postoperative care in these patients to optimise the outcomes.

**Preoperative Considerations:**

Preoperative planning for EK should be considered for all surgical glaucoma cases. Collaboration with a glaucoma specialist is often required to adjust the glaucoma management plan and to optimise the outcomes.

- Patients with pre-existing glaucoma have a higher risk of experiencing elevated IOP following a DMEK procedure. Therefore, establishing good intraocular pressure (IOP) control prior to surgery is crucial [83]. This includes determining the patient’s tolerance to drops and whether they are a steroid responder in order to better manage the postoperative treatment regime safely and effectively.
- In patients with tube implants, the position of the tube must be assessed. In case of anteriorly placed tubes or tubes with a large portion of the tip being in the anterior chamber, the tube may need trimming or repositioning before endothelial keratoplasty [27,84]. This can be performed intraoperatively at the time of endothelial keratoplasty.

**Intraoperative considerations:**

- The main incision should be made in a way that avoids filtering blebs, tubes, or other existing surgical incisions. Additionally, it is advisable to preserve the superior conjunctiva to accommodate any future glaucoma surgeries [85].
- Adjustments of donor graft size might be needed based on the white to-white and the location of the drainage device to avoid any contact between the drainage tube and the Descemet membrane [85].
- Adjustment of the shape of the graft is described for both DMEK and DSAEK to avoid tissue overlap in the area of the drainage tube. For DMEK, using three-quarters of a large (11–12 mm) graft has been recommended [86] and for DSAEK a peripheral notch, made by a 2 mm skin biopsy punch, can be made in the transplant tissue to overlay the silicone tube [87].
- Adequate air fill is important for graft attachment, while avoiding excessive pressure is essential to protect the optic nerve in glaucomatous eyes.
- Synechiae management: Trabeculectomy and tube surgeries can lead to anterior or posterior synechiae, which can hinder graft unfolding and centration. These adhesions should be lysed to allow the DMEK graft to unfold and position properly in the anterior chamber [88]. Synechiae can be released at the beginning of the surgery with visco-dissection, followed by careful removal of the viscoelastic.
- Descemetorhexis—in patients with tubes, descemetorhexis under viscoelastic may block the tube and significantly impair the glaucoma control. To avoid blockage of the glaucoma drainage device, a technique has been described that used pressurised air infusion within the AC to help perform descemetorhexis [89]. Additionally, this technique helps maintain a stable anterior chamber and prevent bleeding during peripheral iridectomy (PI) and synechiolysis, reducing fibrin formation in the anterior chamber, which can complicate unfolding of the graft.
- Air or gas injection: In trabeculectomy or tube patients, the filtering bleb can allow rapid leakage of the air bubble used to support graft adhesion. Therefore, surgeons may use a longer-lasting gas such as sulphur hexafluoride (SF6) instead of air, which helps maintain graft adhesion for a longer period [90]. However, this remains controversial, since the risk of IOP spike is higher with SF6. SF6 should be used with caution in patients with advanced glaucoma as the outflow system is abnormal [85]. Instead, with additional air injection a full air-filled anterior chamber is often achievable in these eyes [91].

**Postoperative considerations:**

- Rebubbling and graft detachment: The risk of graft detachment is higher in patients with previous glaucoma surgery [92]. Hence, graft rebubbling may be needed more frequently. As mentioned previously, either SF6 or overfilled air injection to the anterior chamber are recommended.
- Post-EK elevated IOP: The mechanisms of raised intraocular pressure (IOP) after DMEK include reverse pupillary block in the early postoperative period, and steroid response, persistent inflammation, and peripheral anterior synechia in the late phase [73]. Early recognition and treatment of raised IOP is important after DMEK. A prophylactic peripheral iridectomy is recommended to prevent reverse pupillary block. Patients should posture face-up and be evaluated in the early postoperative period, as patients may be asymptomatic despite raised IOP. To reduce the risk of a steroid response, weaker steroids may be prescribed [83].

## 6. Conclusions

Both DMEK and DSAEK are feasible surgical options for patients with existing glaucoma. Though statistical results are comparable in graft detachment, rejection, survival, and impact on IOP and glaucoma control, DMEK presents potential distinct advantages over DSAEK. DMEK provides better visual outcomes and faster visual recovery than DSAEK and a lower risk of immunological rejection. Additionally, DMEK requires fewer topical corticosteroid drops postoperatively, reducing the risk of steroid-induced increases in intraocular pressure.

## 7. Limitations

This topic is significantly limited by a lack of prospective studies and randomised controlled trials. The studies reviewed are retrospective in nature, with the majority being non-comparative, introducing bias to the data being compared for the purpose of this review. Additionally, data reported on glaucoma patients are often grouped. Surgical glaucoma patients, for example, encompass those who have had a trabeculectomy, minimally invasive glaucoma surgery (MIGS), or glaucoma drainage device, and subgroup analysis on these distinct patients is often not presented. There may be significant differences when looking at these subgroups, which can help to guide treatment options. Further literature is required, ideally in a randomised controlled trial, to determine whether DMEK or DSAEK should be the method of choice in patients with various degrees of glaucoma severity.

## Figures and Tables

**Table 3:** Risk factors for IOP elevation and strategies to mitigate them.

Risk Factors for IOP Elevation Following EK	Strategies to Mitigate These Risks
Pre-existing glaucoma or ocular hypertension	Careful patient selection, liaising with glaucoma team and preoperative IOP optimisation
Pupillary block	Perform preoperative peripheral iridotomy or administer postoperative cycloplegic eye drops
Retained viscoelastic or air/gas overfill	Judicious removal of all viscoelastic material. Slight decompression of the anterior chamber after 10 min full air fill
Graft oversizing or graft displacement	Controlled graft insertion and positioning, trimming of GDD to keep away from graft
Peripheral anterior synechiae and angle compromise from surgical manipulation	Cautious surgical technique to minimise angle trauma and synechiae formation Peripheral iridotomy
Postoperative inflammation	Close postoperative monitoring
Steroid response	Early tapering for steroid responders Consider loteprednol
